# Successful treatment of metastatic hepatic epithelioid hemangioendothelioma with thalidomide: a case report

**DOI:** 10.1186/1752-1947-4-413

**Published:** 2010-12-22

**Authors:** Claire Raphael, Emma Hudson, Leslie Williams, Jason F Lester, Philip M Savage

**Affiliations:** 1 Department of Medical Oncology, Charing Cross Hospital, Imperial College Healthcare NHS Trust London W6 8RF, UK; 2 Department of Clinical Oncology, Velindre Hospital, Cardiff CF14 2TL, UK; 3 Department of Radiology, Velindre Hospital, Cardiff CF14 2TL, UK

## Abstract

**Introduction:**

Hepatic epithelioid hemangioendothelioma is a rare malignancy arising from the vascular endothelial cells within the liver. Historically, the disease is characterized as being poorly responsive to both chemotherapy and radiotherapy, with liver resection or transplantation the treatment of choice when feasible. For patients with advanced disease, reports of long-term therapeutic benefits from conventional cytotoxic treatments are very limited. Owing to the rarity of this malignancy, there is no structured therapeutic research, but a small number of cases have been reported to respond well to treatment with inhibitors of angiogenesis. Thalidomide was originally developed as an anti-emetic but is a potent inhibitor of vascular neogenesis, and could offer potential in the treatment of hepatic epithelioid hemangioendothelioma by blocking the proliferation of the malignant vascular endothelial cells.

**Case presentation:**

We describe the case of a Caucasian British woman who presented at the age of 53 years with a hepatic mass, malignant lymphadenopathy and pulmonary metastases, which were confirmed as hepatic epithelioid hemangioendothelioma on biopsy. After unproductive treatment with interferon, our patient was started on thalidomide 400 mg daily. She has been successfully managed on this therapy for the past seven years, and has remained asymptomatic, with radiologically stable disease and minimal treatment-related side effects.

**Conclusion:**

At present, there is no standard therapy for advanced hepatic epithelioid hemangioendothelioma. Our case supports the role for thalidomide and potentially other inhibitors of vascular neogenesis in the treatment of patients with metastatic hepatic epithelioid hemangioendothelioma.

## Introduction

Originally described in 1982, hepatic epithelioid hemangioendothelioma (HEH) is a rare neoplasm arising from the vascular endothelial cells of the liver [1] The incidence is estimated at less than one case per million in the population, with the diagnosis occurring across a wide age range and with a male:female ratio of 2:3 [2-5].

The optimal management of patients with metastatic disease is yet to be established. When the disease remains confined to the liver, hepatic resection or orthotopic liver transplantation are the treatments of choice [6]. Because of the rarity of the condition, the effects of treatment are difficult to assess in a systematic manner but several case reports have described benefits for treatment with a range of therapies including chemotherapy [7] and interferon [8]. However, despite these reported cases, HEH remains a difficult condition to manage, without any apparent routine benefit from various chemotherapy, immunotherapy or radiotherapy approaches [3].

Thalidomide was first introduced as a treatment for morning sickness in Europe in the 1950 s, but was withdrawn after its severe teratogenic effects became apparent [9]. More recently, the anti-vasculogenic, immunomodulatory and anti-inflammatory properties of thalidomide have shown clinical benefits in malignancies including multiple myeloma, for which it is a licensed therapy, and also in the experimental therapy of prostate cancer and renal cell carcinoma [10].

Currently, data on the use of thalidomide in the treatment of HEH are limited to a small number of case reports demonstrating significant clinical benefits for its use either as monotherapy [11] or in combination with other anti-angiogenic agents [12].

In this report, we describe a further case of HEH treated with thalidomide. The drug has proven to be successful therapy for a patient with advanced metastatic disease extending in excess of seven years, without significant treatment-related toxicity.

## Case presentation

**Figure 1:**
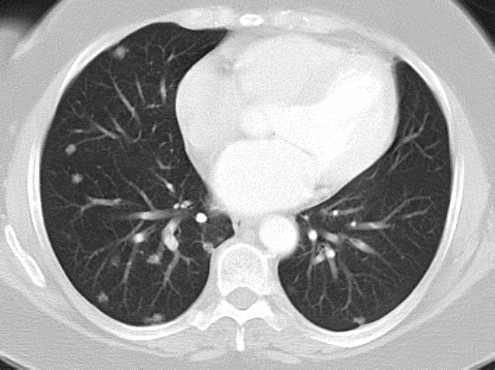
**Computed tomography scan of the thorax performed at presentation in 2002 demonstrating multiple pulmonary metastases**.

**Figure 2:**
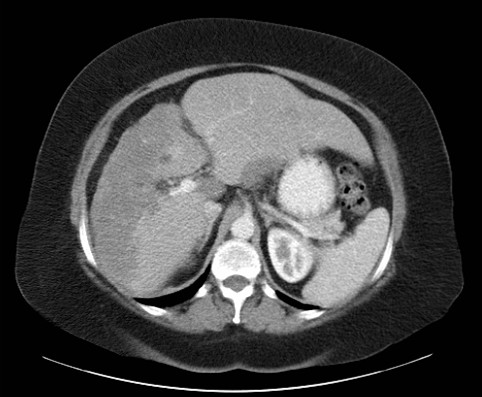
**Abdominal computed tomography scan from 2002 showing a large tumour in the liver that was confirmed on biopsy as hepatic epithelioid hemangioendothelioma**..

A 53-year-old Caucasian British woman originally presented to her local hospital in 2002 with shortness of breath secondary to atrial fibrillation. During the admission, chest radiography revealed widespread pulmonary nodular infiltrates, and a subsequent computed tomography (CT) scan confirmed the presence of widespread pulmonary metastases, most marked in the lower lung fields (Figure 1). The CT scan also demonstrated extensive retroperitoneal and para-aortic lymphadenopathy and hepatic abnormalities consistent with metastases, but no obvious primary site for the disease (Figure 2).

Before admission, our patient had no symptoms of liver dysfunction and her medical history was otherwise unremarkable. She had no family history of malignancy. Routine haematology, clotting, serum biochemistry and liver function tests, hepatitis screen and tumour markers were all within normal limits. After the standard medical management for atrial fibrillation was completed, a CT-guided biopsy of the predominant hepatic lesion was performed.

Histological examination of the biopsy confirmed the presence of an infiltrating tumour with pleomorphic elongated cells that stained positively with the vascular markers Factor VIII, CD31, and CD34. These morphological and immunohistochemical features were consistent with the diagnosis of HEH. In the presence of disseminated disease, surgical management was not indicated, and our patient was referred to the oncology team for ongoing management.

In response to radiological evidence of disease progression, first-line therapy with interferon was commenced in accordance with the dosing recommendations published in an earlier case report [8]. A CT scan performed after three months therapy demonstrated stable disease; however, as our patient was experiencing significant interferon-related side effects, the treatment was discontinued. After a six-month break from therapy, there was evidence of disease progression within the spleen seen on an updated CT scan, and the decision was taken to explore treatment with thalidomide.

**Figure 3:**
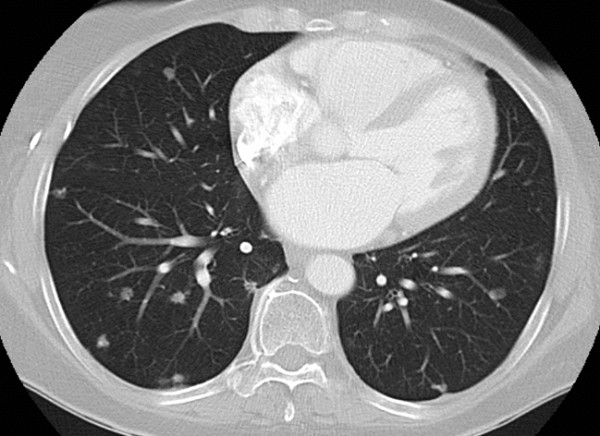
**Computed tomography scan of the thorax performed after seven years of treatment showing the lack of any significant disease progression during thalidomide therapy**.

**Figure 4:**
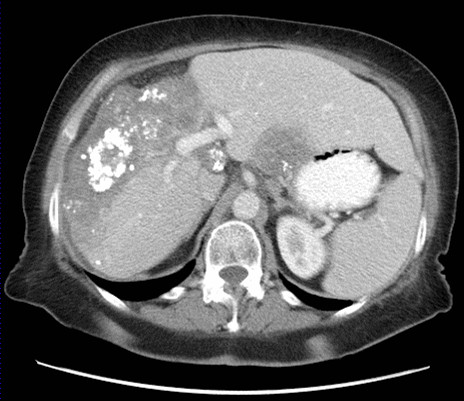
**Computed tomography scan of the abdomen performed in 2009 demonstrating an unchanged size of the liver lesion over the seven-year period but with the development of calcification**.

Starting initially at 100 mg per day, the dose of the drug was increased at weekly intervals up to 400 mg. Treatment was well tolerated and currently remains at 400 mg daily, more than seven years later. During this period, our patient has had regular restaging by CT scans; the disease has remained stable in the lungs and liver by Recist criteria, but with the development of calcification within the predominant liver lesion (Figure 3 Figure 4). At present, our patient continues to have no symptoms of the disease, and throughout the course of treatment has not had any treatment-related toxicity that has required either hospitalization or dose reduction.

## Conclusion

We report a case of metastatic HEH effectively treated using thalidomide, To the best of our knowledge, this is the third report to describe successful treatment of this rare disease using this agent. In contrast to an earlier report [11], we did not find any major reduction in disease bulk with thalidomide treatment; however, our patient has remained clinically and radiologically stable over a period in excess of seven years. More recently, the case of a patient treated with lenalidomide, a derivative of thalidomide, has been described, reporting long-term benefit in a patient with HEH progressing after earlier chemotherapy treatment [13].

Although the evidence in support for treatment of metastatic HEH with thalidomide or lenalidomide is limited to case reports, it is unlikely, because of the rarity of this condition, that any formally structured clinical research can readily be performed. This apparent activity combined with the modest side-effect profile of thalidomide or lenalidomide, and the lack of any other standard therapy makes a strong case for considering these drugs as first-line therapy of metastatic HEH.

In summary, we successfully used thalidomide in the treatment of HEH with widespread pulmonary, hepatic and retroperitoneal metastases, with stable disease after seven years of follow-up. Thalidomide is a low toxicity agent that acts as an inhibitor of vascular neogenesis, and seems both an intuitive and clinically supported choice of agent for treatment of this malignancy when surgical management is not appropriate.

## Competing interests

The authors declare that they have no competing interests

## Consent

Written informed consent was obtained from the patient for publication of this case report and any accompanying images. A copy of the written consent is available for review by the Editor-in-Chief of this journal.

## Authors' contributions

All the authors have read and approved the final version of this manuscript. CR, EH and PS assembled the clinical data and wrote the paper. EH, LW, JL and PS were involved in the clinical care. PS is the corresponding author.
